# Evaluation of Consumer Understanding of Different Front-of-Package Nutrition Labels, 2010–2011

**DOI:** 10.5888/pcd9.120015

**Published:** 2012-09-20

**Authors:** Christina A. Roberto, Marie A. Bragg, Marissa J. Seamans, Regine L. Mechulan, Nicole Novak, Kelly D. Brownell

**Affiliations:** Author Affiliations: Marie A. Bragg, Nicole Novak, Kelly D. Brownell, Rudd Center for Food Policy and Obesity, Yale University, New Haven, Connecticut; Marissa J. Seamans, School of Global Public Health, University of North Carolina at Chapel Hill, Chapel Hill, North Carolina; Regine L. Mechulan, Cornell University, Ithaca, New York.

## Abstract

**Introduction:**

Governments throughout the world are using or considering various front-of-package (FOP) food labeling systems to provide nutrition information to consumers. Our web-based study tested consumer understanding of different FOP labeling systems.

**Methods:**

Adult participants (N = 480) were randomized to 1 of 5 groups to evaluate FOP labels: 1) no label; 2) multiple traffic light (MTL); 3) MTL plus daily caloric requirement icon (MTL+caloric intake); 4) traffic light with specific nutrients to limit based on food category (TL+SNL); or 5) the Choices logo. Total percentage correct quiz scores were created reflecting participants’ ability to select the healthier of 2 foods and estimate amounts of saturated fat, sugar, and sodium in foods. Participants also rated products on taste, healthfulness, and how likely they were to purchase the product. Quiz scores and product perceptions were compared with 1-way analysis of variance followed by post-hoc Tukey tests.

**Results:**

The MTL+caloric intake group (mean [standard deviation], 73.3% [6.9%]) and Choices group (72.5% [13.2%]) significantly outperformed the no label group (67.8% [10.3%]) and the TL+SNL group (65.8% [7.3%]) in selecting the more healthful product on the healthier product quiz. The MTL and MTL+caloric intake groups achieved average scores of more than 90% on the saturated fat, sugar, and sodium quizzes, which were significantly better than the no label and Choices group average scores, which were between 34% and 47%.

**Conclusion:**

An MTL+caloric intake label and the Choices symbol hold promise as FOP labeling systems and require further testing in different environments and population subgroups.

## Introduction

The Nutrition Labeling and Education Act of 1990 requires most packaged foods in the United States to display the Nutrition Facts panel (1). However, since the law’s enactment, the US food and beverage industry has initiated many front-of-package (FOP) nutrition labeling systems to provide consumers with key nutrition information that can be quickly and easily interpreted (2). The industry also released a new FOP labeling system called Facts Up Front that includes information about calories, saturated fat, sodium, sugars, and other nutrients (3).

Different FOP symbols are in use outside the United States. Two prominent ones are the Traffic Light (TL) food labeling system developed by the United Kingdom’s Food Standards Agency (UK FSA) and the Choices symbol, adopted by countries such as the Netherlands and Poland (4,5). The TL system highlights amounts of total fat, saturated fat, sugar, and sodium in foods, and the Choices symbol appears on products that meet dietary guidelines developed by an international scientific committee (5).

The existence of various FOP labeling systems prompted the US Food and Drug Administration (FDA) to undertake an initiative (6) to evaluate these systems. As part of this initiative, the Institute of Medicine (IOM) prepared 2 reports that provide science-based recommendations for an optimum labeling system (7,8). However, few studies of FOP labeling systems in US populations have been conducted, making it difficult to draw conclusions about the best system. Our study was designed to determine which of several approaches to FOP labeling would be most likely to enable consumers to identify the healthiest food options within the same food category and would promote accurate understanding of the nutritional composition of packaged foods. We examined 4 labels in our study, including the Choices symbol and 3 different versions of the Multiple Traffic Light (MTL) system (4), a calories-per-serving label system that indicates whether a product contains high, medium, or low amounts of 3 nutrients that should be consumed in limited quantities: saturated fat, sodium, and sugar.

## Methods

### Participant selection

We recruited participants through the Yale School of Management’s E-lab, an online database of approximately 15,000 US adults who enrolled to receive invitations for online research studies. E-lab is advertised via websites, e-mail, and in-person research studies and has been used to study other food and weight-related research questions (9). Our study was advertised in the fall of 2010 as a 20-minute survey on nutrition information of packaged foods, and we conducted our study in January and February 2011. As an incentive to participate, enrollees were told they had a 1 in 10 chance to win a $25 gift card.

Of 15,000 E -lab enrollees contacted, 527 agreed to participate in our study. Of these, we excluded 14 for completing less than 25% of the survey, and we excluded 33 for providing nonsense answers or for spending less than 5 minutes to finish the survey. The final participant sample was 480, 64% of whom were female. The mean age was 36 years (standard deviation, 13 y; range, 18–76 years), and the mean body mass index (BMI) was 27.3 (7.4) kg/m^2^. The study groups did not differ significantly by proportion of people excluded (*P* = .50), age, BMI, education level, annual income, or reported efforts to lose weight (Table 1).

**Table 1:** Consumer Understanding of Front-of-Package Nutrition Labels: Participants’ Sociodemographic Information (N = 480)^a^, 2010–2011

Characteristic	Front-of-Package Labels Evaluated					
	No Label Control (n = 99)	Choices (n = 98)	MTL (n = 98)	MTL+Caloric Intake (n = 90)	TL+SNL (n = 95)	*P* ^b^
**Age, y, mean (SD)**	36 (12)	36 (12)	38 (15)	36 (13)	35 (14)	.54
**Body mass index (kg/m^2^), mean (SD)**	26.8 (7.5)	27.0 (7.1)	27.3 (6.5)	28.6 (7.8)	26.5 (7.7)	.35
**Influence of labels^c^, mean (SD)**	6.5 (2.2)	6.2 (2.2)	6.0 (2.2)	6.5 (2.0)	6.1 (2.0)	.46
**Sex**						
Female	59 (63)	61 (65)	60 (63)	56 (64)	59 (66)	.99
Male	34 (37)	33 (35)	36 (38)	31 (36)	31 (34)	
**Race/ethnicity**						
White	66 (71)	76 (81)	76 (79)	72 (83)	67 (74)	.49
Asian	16 (17)	10 (11)	7 (7)	11 (13)	11 (12)	
Hispanic	3 (3)	2 (2)	3 (3)	2 (2)	6 (7)	
African American	7 (8)	3 (3)	8 (8)	2 (2)	5 (6)	
Other	1 (1)	2 (2)	2 (2)	0	1 (1)	
**Educational level**						
<High school diploma	10 (11)	10 (11)	6 (6)	7 (8)	9 (10)	.80
Some college	19 (20)	27 (29)	26 (27)	24 (28)	30 (33)	
2-year college degree	12 (13)	7 (7)	13 (14)	10 (12)	7 (8)	
4-year college degree	36 (39)	31 (33)	34 (35)	31 (36)	35 (39)	
Graduate degree	16 (17)	19 (20)	17 (18)	15 (17)	9 (10)	
**Annual income, $**						
<15,000	7 (8)	6 (6)	9 (9)	6 (7)	9 (10)	.21
16,000-44,999	21 (23)	31 (33)	20 (21)	28 (32)	27 (30)	
45,000-89,999	44 (47)	34 (36)	38 (40)	38 (44)	42 (47)	
90,000-150,000	11 (12)	14 (15)	18 (19)	12 (14)	18 (20)	
>150,000	10 (11)	9 (10)	11 (11)	3 (3)	3 (3)	
**Currently trying to lose weight**						
Yes	40 (43)	42 (45)	41 (43)	49 (56)	41 (46)	.34
No	53 (57)	52 (55)	55 (57)	38 (44)	49 (54)	

Abbreviation: SD, standard deviation.

^a^ Study groups were no label (control), Choices (Choices symbol), MTL (multiple traffic light), MTL+caloric intake (multiple traffic light plus daily calorie recommendation icon), TL+SNL (traffic light plus specific nutrients to limit). For all categorical variables, values are presented as n (%); percentages were calculated according to number of participants who completed each question, which varied among questions.

^b^ *P* value is for univariate analysis of variance (continuous variables) or χ^2^ test (categorical variables).

^c^ Measured on 9-point Likert scale.

After providing informed consent, participants viewed an online survey of food products that displayed 5 different FOP labels. The Yale University Human Subjects Committee approved this study.

### Front-of-package labels

**Figure 1:**
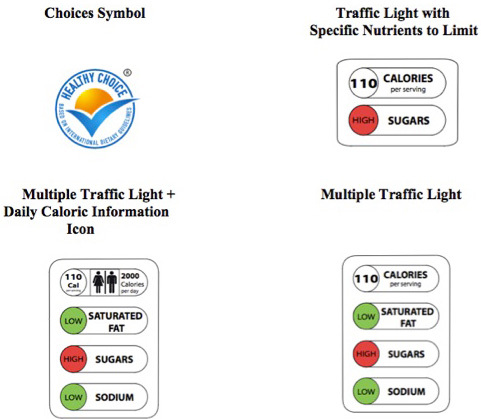
Front-of-package food label symbols tested in evaluation of consumer understanding of different front-of-package nutrition labels.

Participants were assigned to 1 of 5 groups: a control group that viewed food products with no FOP label and 4 groups that viewed products that included 1 of 4 different FOP labels (Figure 1):

**Table:** 

The figure contains 4 colored icons representing 4 front-of-package labels:
1. Multiple traffic lights (MTL) label: A calorie-per-serving label with a traffic light (TL) symbol with High, Med, or Low superimposed on the red, yellow, and green lights, respectively, indicating that the product contains high, medium, or low amounts of 3 nutrients whose consumption should be limited: saturated fat, sodium, and sugar.
2. MTL plus a daily caloric intake label (MTL+ caloric intake): Same as MTL but with the addition of male and female icons accompanied by the words “2000 calories per day.”
3. Traffic light with specific nutrients to limit label (TL+SNL): A calories-per-serving label plus the TL symbol with the text High, Med, or Low superimposed on the red, yellow, and green lights, respectively, indicating that the product contains high, medium, or low amounts of nutrients whose consumption should be limited on the basis of nutrients of concern in that particular food category. Some food types contained more than 1 nutrient to limit, in which case the label included a TL for each of those nutrients. We consulted 2 nutrition experts to determine specific nutrients to limit for each food category and used their suggestions in the test label.
4. Choices logo (Choices): The Choices symbol in use worldwide consists of a check mark and the words “Healthy Choice.” The symbol indicates that products contain lower levels of sodium, sugar, saturated fat, trans fat, and calories and greater amounts of dietary fiber relative to similar products in the same category.

### Survey procedure

**Figure 2:**
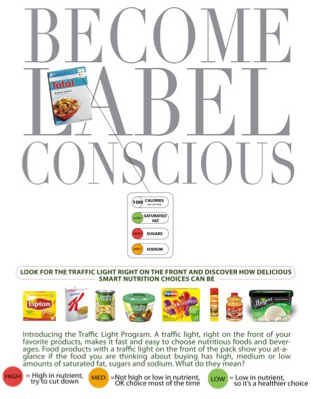
Sample public service advertisement explaining interpretation of front-of-package labeling system.

Participants in each of the 5 groups viewed a public service advertisement (PSA) online (Figure 2) that was modeled after a food industry advertisement for a previous FOP labeling program called Smart Choices (10). The PSA that each of the 5 groups viewed included the words “become label conscious.” PSAs for participants in the 4 label groups included instructions on how to interpret the labeling system the participant was about to see. Participants in the no-label control group viewed a PSA nearly identical to those viewed by the other 4 groups but without label interpretation instructions. We included a PSA in the survey because an FOP initiative would likely be accompanied by such educational efforts, and the food industry pledged to spend $50 million on a consumer education campaign for Facts Up Front.

After viewing the PSA, participants took a 15-item quiz asking them to identify what they considered the healthier of 2 products and were instructed to rate 7 products (Nesquik Chocolate Milk, McVitie’s Toasting Waffles, Health Valley Apple Cereal Bars, I Can’t Believe It’s Not Butter! Mediterranean Blend, Pfeiffer Dressing California French, Orville Redenbacher Smart Pop Kettle Corn Microwave, and Campbell’s Chunky Grilled Sirloin Steak with Hearty Vegetables) for perceptions of nutrient levels, healthfulness of the product, how good they thought the product would taste, and how likely they would be to buy the product. Product presentation order was not randomized.

### Product selection and nutrition classification

US brand name and lesser-known UK and Canadian products were selected from 8 food product categories. Participants viewed the front of each product, and an enlarged version of the FOP label appeared adjacent to the product to ensure its visibility. All health claims such as “reduced fat” were removed from these product images.

Product nutrition information was obtained from the Nutrition Facts panel on the package or from food manufacturer websites. The nutrient profile model (NPM) was used to generate a numerical score for each food and beverage (11). The NPM adds points for negative nutrients (eg, saturated fat, sugar, sodium) and calories and subtracts points for positive nutrients (fiber, protein) and percentage of fruit, vegetables, or nuts the product contains. The packaged foods in this study contained minimal amounts of fruit, vegetables, and nuts; therefore, these foods were not factored into the model. Foods with a score of less than 4 and beverages with a score of less than 1 are considered healthy. The UK government has used NPM to identify foods that can be advertised during children’s television programming, and the model has been validated (12–14).

#### Traffic light labels

The TL labels (MTL, MTL+caloric intake, TL+SNL) had red, yellow, and green circles labeled high, medium, and low indicating amounts of nutrients in the products. Percentage daily values were not included in the labels because research indicates that consumers find these values confusing (4,15–18). The classification of problem nutrients as high, medium, or low was based on the UK FSA’s TL criteria (11).

#### Choices label

The Choices symbol appears only on products meeting nutrition standards developed by an independent scientific body (5). To ensure that the nutrition information that served as the basis of the TL labels and Choices symbol in this study were the same, the NPM score was used to determine whether a product qualified for a Choices symbol. This enabled us to test the FOP label rather than the nutrition criteria it represented. When participants were asked to identify the healthier of 2 products, 5 of the 15 comparisons included 2 products that did not qualify for the Choices logo. We included these 5 to ensure that comparisons reflected a range of real-world food decision-making scenarios given that many packaged foods would not qualify as healthy based on these criteria. Similarly, when participants were asked to provide ratings of 7 individual products, only 1 of the 7 products qualified for a Choices logo.

### Evaluating participant nutrient understanding and product perceptions

Participants viewed a series of 15 food comparisons and selected the healthier of 2 products in the same food category presented side-by-side. Both products had the same type of FOP label describing the nutrition information for that product. Participants’ product selections were coded as correct if the product had a lower NPM score, indicating it was a healthier product. Correct answers on each quiz question were summed to produce a total percentage-correct score. Correct answers for each individual product comparison were also examined across study groups.

Participants assessed whether the individual products had high, medium, or low levels of saturated fat, sugar, and sodium. Correct answers were based on the UK FSA TL nutrition criteria, which were the basis for the “high, medium, low” on the TL labels (12). Total percentage-correct scores were calculated for each quiz.

Participants estimated calories per serving for the individual products. We calculated the absolute difference between the actual and the participant-estimated calories per serving for each product and averaged the discrepancies across all 7 products. Participants also estimated the recommended number of calories a normal-weight adult should consume daily. Answers between 1,500 and 2,500 calories per day were considered correct.

Participants rated how healthy each product was, how good they thought it would taste, and how likely they would be to buy the product for themselves or their children. They used a 9-point Likert scale for their ratings. Each set of ratings was averaged across all 7 products. Because Campbell’s Chunky Grilled Sirloin Steak with Hearty Vegetables was the only product that qualified for the Choices symbol, analyses were repeated to examine this product separately. At the end of the survey, participants provided demographic information and indicated whether they were currently trying to lose weight and the degree to which nutrition labels generally influence their food choices, registered on a 9-point Likert scale.

### Statistical analyses

Data analysis was performed using SPSS 18.0 (SPSS Inc, Chicago, Illinois). Continuous study outcomes were compared by using univariate ANOVA followed by post-hoc Tukey tests. We used χ^2^ tests to examine categorical outcome variables. Eta squared effect sizes were calculated for ANOVA analyses and interpreted based on Cohen’s guidelines (small = 0.01, medium = 0.06, large = 0.14). Cohen’s *d* was also calculated as a measure of effect size for post-hoc group comparisons and interpreted based on Cohen’s guidelines (small = 0.2, medium = 0.5, large = 0.8). Results for primary outcomes were considered significant if unadjusted *P* values were less than a Bonferroni-corrected α of .005. In addition, a Bonferroni-corrected α of .003 was applied to secondary analyses examining correct answers for individual product comparisons. Significance for all other tests was assessed at an α of .05.

## Results

### Study outcomes

#### Healthier product quiz

Participant scores for the healthier product quiz varied significantly among groups (Table 2). The Choices group outperformed the no label group (Cohen’s *d* = 0.40, *P* = .005) as did the MTL+caloric intake label group (*d* = 0.62, *P* < .001). The TL+SNL group had fewer correct answers than the Choices group (*d* = 0.63, *P* < .001), the MTL group (*d* = 0.69, *P* = .001), and the MTL+caloric intake group (*d* = 1.06, *P* < .001).

**Table 2:** Consumer Understanding of Front-of-Package Nutrition Labels: Percentage Correct for Healthier Product Quiz Items (N = 480)^a^, 2010–2011

Product Comparison (Nutrient Profile Model Score)	No Label (n = 99)	Choices (n = 98)	MTL (n = 98)	MTL+Caloric Intake (n = 90)	TL+SNL (n = 95)	χ^2^	*P* ^b^
**Beverages**							
Vintage Seltzer Water (0)^b^	95	95	97	90	93	4.8	.31
Walgreens Refreshing Cola (2)							
**Breads and grains**							
Wonder Classic White Bread (5)	92	93	33	35	37	157.5	<.001^c^
Arnold’s Bread, Soft Honey Wheat (0)^b^							
Kingmills 50/50 Bread with Omega 3 (−4)^b^	57	70	83	78	73	28.5	<.001^c^
Auntie Hattie’s Potato Bread (11)							
**Cereals**							
Kellogg’s Mini-Wheats Unfrosted Bite Size (−6)^b^	31	44	23	42	12	36.2	<.001^c^
General Mills Cheerios (0)							
Meijer Frosted Bite Size Shredded Wheat (−2)^b^	26	43	55	49	13	54.5	<.001^c^
Uncle Sam Toasted Whole Wheat Flakes and Flaxseed (8)							
**Condiments and dressings**							
Kraft Tuscan House Italian Dressing (20)^b^	91	87	93	89	91	9.9	.04
Kraft Buttermilk Ranch Dressing (26)							
Morehouse Mustard (10)^b^	96	89	94	87	91	5.4	.25
Duke’s Mayonnaise (23)							
**Desserts**							
Klondike Original Ice Cream Bar (17)	69	86	94	88	93	60.0	<.001^c^
Breyer’s Smooth and Dreamy Fat Free Ice Cream, Creamy Vanilla (-1)^b^							
Tesco Vanilla Ice Cream (7)^b^	75	71	96	90	92	66.6	<.001^c^
Mangum Classic Vanilla Ice Cream Bar (18)							
**Fats, oils, and spreads**							
I Can’t Believe It’s Not Butter! Spray (0)^b^	20	36	85	72	67	133.8	<.001^c^
Mazola Olive Oil (19)							
Imperial Margarine (24)	41	39	3	0	1	124.0	<.001^c^
Utterly Butterly Spread (15)^b^							
**Snacks**							
Orville Redenbacher Original Kernel (−7)^b^	91	96	96	89	92	9.3	.05
Kraft Macaroni and Cheese Baked Crackers (21)							
Fudges Cheddar Wafers (26)	60	56	90	86	85	78.0	<.001^c^
Butterkist Salted Popcorn (20)^b^							
**Soups**							
Progresso Soup, Traditional Chicken Noodle (2)^b^	84	76	93	90	90	35.4	<.001^c^
Campbell’s Condensed Soup, Chicken Noodle (3)							
Dinty Moore Scalloped Potatoes and Ham (5)	79	85	8	4	7	289.7	<.001^c^
Stagg Classic Chili (0)^b^							

^a^ Study groups were no label (control), Choices (Choices symbol), MTL (multiple traffic light), MTL+caloric intake (multiple traffic light plus daily calorie recommendation icon), TL+SNL (traffic light plus specific nutrients to limit.

^b^ Represents the “correct” answer (based on the product with the lower Nutrient Profile Model [NPM Score] on a healthier product quiz that asked participants to choose the healthier of 2 products in the same food category (11). The NPM adds points for negative nutrients (eg, saturated fat, sugar, sodium) and calories and subtracts points for positive nutrients (fiber, protein). Foods with a score of less than 4 and beverages with a score of less than 1 are considered healthy.

^c^ Significant based on a χ^2^ test comparing front-of-package label groups and a Bonferroni corrected α level of .003.

#### Saturated fat, sodium, and sugars quizzes

The label groups differed significantly on the saturated fat quiz. All 3 TL groups significantly outperformed the no label and Choices groups (*P* < .001 for all TL comparisons). Cohen’s *d* effect sizes for the control group versus the TL groups were as follows: MTL, *d* = 3.09; MTL+caloric intake, *d* = 3.70; and TL+SNL, *d* = 1.90. The 2 MTL groups had significantly higher scores than all other groups. There were also significant differences among study groups on the sugars and sodium quizzes, which were similar to the pattern of findings for the saturated fat quiz.

#### Accuracy of calories-per-serving estimate

The 4 label groups significantly differed on ability to estimate calories per serving (Table 3). All 3 TL groups led to more accurate calories-per-serving estimates compared with the Choices and no-label groups: MTL, *d* = 0.73; MTL+caloric intake, *d* = 0.77; TL+SNL, *d* = 0.75; (*P* < .001 for all comparisons). Only 17% of participants provided an incorrect estimate of recommended daily caloric intake, which did not differ across group (χ^2^_4_) = 7.0, *P* = .14).

**Table 3:** Consumer Understanding of Front-of-Package Nutrition Labels: Food and Beverage Ratings^a^ (N = 480)^b^, 2010–2011

Outcome	No Label, Control Group(n = 99)	Choices (n = 98)	MTL (n = 98)	MTL+ Caloric Intake (n = 90)	TL+SNL (n = 95)	*P^c^*	η^2 d^
Healthier product quiz	67.8 (10.3)	72.5 (13.2)^e,i^	71.0 (7.7)^i^	73.3 (6.9)^e,i^	65.8 (7.3)^f,g,h^	<.001	.002
Saturated fat quiz	33.6 (16.3)	35.7 (15.9)^g,h,i^	90.7 (20.4)^e,f,i^	92.3 (15.1)^e,f,i^	64.9 (16.5)^e,f,g,h^	<.001	.131
Sugars quiz	47.2 (19.6)	41.9 (19.4) ^g,h,i^	90.0 (20.7) ^e,f,i^	92.6 (15.5)^e,f,i^	77.2 (19.5)^e,f,g,h^	<.001	.080
Sodium quiz	42.6 (20.7)	47.1 (21.1) ^g,h,i^	93.5 (16.4) ^e,f,i^	94.1 (15.6)^e,f,i^	72.4 (15.7) ^e,f,g,h^	<.001	.085
Absolute difference in estimated and actual calories per serving	115.1 (171.8)	88.9 (105.4) ^g,h,i^	15.2 (88.1) ^e,f^	16.9 (57.6)^e,f^	14.8 (78.5)^e,f^	<.001	.118
Healthfulness^j^	4.4 (1.3)	4.6 (1.4) ^g,h,i^	3.8 (1.2)^e,f^	3.6 (1.1)^e,f^	3.8 (1.2)^e,f^	<.001	.009
Taste^j^	5.8 (1.4)	5.3 (1.5)	5.6 (1.4)	5.4 (1.4)	5.5 (1.3)	.16	.001
**Intent to purchase^j^**							
For self	4.1 (1.5)	4.1 (1.7)	3.8 (1.4)	3.6 (1.5)	3.7 (1.4)	.12	.002
For children	4.3 (1.7)	4.1 (1.9)	4.0 (1.6)	4.0 (1.7)	4.0 (1.5)	.66	.001

^a^ The 7 products used in this analysis were Nesquik Chocolate Milk, McVitie’s Toasting Waffles, Health Valley Apple Cereal Bars, I Can’t Believe It’s Not Butter! Mediterranean Blend, Pfeiffer Dressing California French, Orville Redenbacher Smart Pop Kettle Corn Microwave, and Campbell’s Chunky Grilled Sirloin Steak with Hearty Vegetable.

^b^ Study groups were no label (control), Choices (Choices symbol), MTL (multiple traffic light), MTL+calorie (multiple traffic light plus daily calorie recommendation icon), TL+SNL (traffic light plus specific nutrients to limit. Table values are mean (SD). The total N varied with the number of participants that completed each question.

^c^ Significant based on univariate ANOVAs comparing front-of-package label groups and a Bonferroni corrected α level of .003.

^d^ Represents the effect size for the overall ANOVA analysis.

^e^ Significantly different from control group (no label), *P* < .05, based on univariate analysis of variance (ANOVA) and post-hoc Tukey tests.

^f^ Significantly different from Choices label, *P* < .05, based on univariate ANOVA and post-hoc Tukey tests.

^g^ Significantly different from MTL label, *P* < .05, based on univariate ANOVA and post-hoc Tukey tests.

^h^ Significantly different from MTL+caloric intake, *P* < .05, based on univariate ANOVA and post-hoc Tukey tests.

^i^ Significantly different from TL+SNL label, *P* < .05, based on univariate ANOVA and post-hoc Tukey tests.

^j^ Measured on a 9-point Likert scale.

#### Perceptions of health, taste, and intent to purchase

The label groups differed significantly on perceptions of product healthfulness (Table 3). Products were viewed as significantly less healthy in the TL groups relative to the Choices (*P* < .001) and no-label groups (*P* < .001). Cohen’s *d* effect sizes for the control group versus the TL groups were as follows: MTL, *d* = .50; MTL+caloric intake, *d* = .75; and TL+SNL, *d* = .53. Perceptions of taste and purchase intent did not significantly differ across label groups.

#### Outcomes for soup product

For the soup product, which was the only individual product that qualified for a Choices symbol, the Choices group performed significantly worse on the sugar quiz (*P* < .001) than the MTL and MTL+caloric intake groups. The Choices group performed better than the control group on estimating saturated fat (*P* < .001), sodium (*P* < .01), and calories (*P* < .004), but performed significantly worse on these outcomes than all 3 TL groups (*P* < .001) (Table 3).

## Discussion

In this study, labels with the MTL+caloric intake and the Choices symbol were most effective for participants in determining which of 2 products was healthier; the TL+SNL labels were the least effective. In 3 instances the Choices group outperformed the MTL and MTL+caloric intake groups in comparing 2 products. In 2 of these instances, the TLs were the same for the products being compared, but neither product had a Choices symbol. In another instance, the healthier product had a Choices symbol and the comparison product did not; however, for those in the TL groups, the healthier food had a red light for saturated fat while its comparison had no red lights. This highlights a potential limitation of the MTL’s focus on individual nutrients, because consumers may be unduly swayed by a red light when the product as a whole is healthier than other products in that category. In addition, when products have the same pattern of lights, consumers understandably have trouble identifying the healthier food. It is possible that the inclusion of nutrient grams on the label would correct this problem, but the additional information may make the overall label more confusing. In contrast, the Choices labeling system made it more difficult for individuals to identify the healthier of 2 generally unhealthy products because the symbol appeared only on products meeting a certain nutrition threshold. Such a labeling scheme might cause consumers to miss a smaller but potentially meaningful shift to healthier products. These findings suggest that both the Choices and MTL+caloric intake labels have limitations but that overall they perform similarly in educating the consumer.

In estimating nutrient amounts, the TL labels strongly outperformed the Choices symbol, which does not provide specific nutrient information. However, the Choices symbol was more effective than no label for estimating saturated fat, sodium, and calories. The performance of the TL groups on the quizzes suggests that the “high, medium, low” text in combination with the traffic light color-coding scheme was effective at conveying nutrient information. Although the different TL symbols led participants to rate unhealthy products as less healthy, they did not negatively influence taste perceptions. However, intention to purchase the products was not influenced by any of the labels, suggesting that FOP labels in general might have limited influence on behavior.

This study has several limitations. Our participants were a convenience sample that responded to an advertisement for a food study and, thus, may have been more educated about nutrition than the general population. In addition, the sample was predominantly white and of a moderate-to-high socioeconomic status (SES). The more complex MTL symbol may have been easier for this better educated sample to understand than for a lower literacy group, which might perform better with a simpler label system. However, a study conducted in the United Kingdom found that labeling the TL symbols with the words high, medium, and low improved understanding among low SES participants (4). Although middle-aged women comprised a large proportion of our study sample, research suggests that 64% of US consumers doing the grocery shopping are women who are, on average, 47 years old (19). In addition, half of participants were overweight or obese and reported a desire to lose weight, suggesting that we studied a group that comprised a target audience for FOP labels. Although we tested a range of common food and beverage products, we did not assess whether participants usually purchase these products. Also, the presentation of products was not randomized, which may have introduced effects that influenced perceptions of different products based on order and label type. Finally, we examined label comprehension but did not assess whether the labels affected actual rather than intended purchasing patterns or consumption.

This is the first study of US consumers to compare the Choices logo with different, untested versions of the MTL label. Putting the recommended daily calories on a label could help place a product’s nutritional value in context, making it easier to identify healthier products. Participants in European focus groups preferred FOP labels that included daily calorie reference values for adults (17). Research on restaurant menu labeling has also shown that including recommended daily calories prevented greater calorie intake after a meal (20). Taken together, these findings suggest that such information on an FOP label could be useful, but one concern is that a single recommended number of calories is not suitable for everyone. However, 2,000 kilocalories is currently used as the basis for percent daily value nutrient calculations, which appear on the Nutrition Facts Panel. The findings from our study have public health implications given the controversy surrounding the industry’s release of Facts Up Front label (21) and recommendations made by the IOM for an interpretive, ordinal symbol that would indicate the nutritional value of a product based on amounts of saturated fat, trans fat, sodium, and sugar (8). The first step in selecting an FOP labeling system is to determine whether a label can be easily understood and used to estimate a product’s nutrition value. The results of our study suggest that an MTL+caloric intake label system is an easy-to-understand system that can help consumers identify healthier products and evaluate the nutrition composition of foods. In addition, our results caution against a more simplified approach to TL labeling that would only highlight specific nutrients to limit in certain food categories and suggest that the MTL+caloric intake label should be part of the national conversation about FOP labeling systems. An MTL scheme in a cafeteria setting has been found to reduce purchases of “red” products and increase purchases of “green” products, suggesting the same might be true for packaged foods, although the cafeteria’s labeling approach provided a color rating of the foods and beverages overall rather than different colors rating different nutrients in the foods and beverages (22). A next step in decision making about FOP labeling is to conduct research that compares multiple systems, such as the Facts Up Front label, the proposed IOM symbol, the Choices system, and an MTL+caloric intake label and to test these labels in diverse populations.
